# Liquorice for pain?

**DOI:** 10.1177/20451253211024873

**Published:** 2021-07-16

**Authors:** Rae F. Bell, Vânia M. Moreira, Eija A. Kalso, Jari Yli-Kauhaluoma

**Affiliations:** (Emerita) Regional Centre of Excellence in Palliative Care, Haukeland University Hospital, Jonas Lies vei 65, Bergen 5021, Norway; Laboratory of Pharmaceutical Chemistry, Faculty of Pharmacy, University of Coimbra, Coimbra, Portugal; Center for Neuroscience and Cell Biology, University of Coimbra, Coimbra, Portugal; Strathclyde Institute of Pharmacy and Biomedical Sciences, University of Strathclyde, Glasgow, UK; Drug Research Program, Division of Pharmaceutical Chemistry and Technology, Faculty of Pharmacy, University of Helsinki, Helsinki, Finland; Department of Pharmacology and SleepWell Research Programme, Faculty of Medicine, University of Helsinki and Department of Anaesthesiology, Intensive Care and Pain Medicine, Helsinki University Hospital, Helsinki, Finland; Drug Research Program, Division of Pharmaceutical Chemistry and Technology, Faculty of Pharmacy, University of Helsinki, Helsinki, Finland

**Keywords:** glycyrrhizin, inflammation, liquorice, pain

## Abstract

Liquorice has a long history of use in traditional Chinese, Ayurvedic and herbal medicine. The liquorice plant contains numerous bioactive compounds, including triterpenes, flavonoids and secondary metabolites, with glycyrrhizin being the main active compound. Liquorice constituents have been found to have anti-inflammatory, antioxidant, antiviral, anticancer, hepatoprotective and neuroprotective properties. In addition, they appear to have antidepressant actions and effects on morphine tolerance. Glycyrrhizin, its metabolite glycyrrhetic (glycyrrhetinic) acid and other liquorice-derived compounds such as isoflavonoids and *trans*-chalcones, exert potent anti-inflammatory effects *via* a wide range of mechanisms including high mobility group box 1 protein (HMGB1) inhibition, gap junction blockade and α_2A_-adrenoceptor antagonism. These properties, together with an increasing body of preclinical studies and a long history of use in herbal medicine, suggest that liquorice constituents may be useful for pain management. Glycyrrhizin is used widely in the confectionary, food and tobacco industries, but has documented adverse effects that may limit clinical use. Whether liquorice plant-derived compounds represent a novel class of analgesics is yet to be established. Having a host of bioactive compounds with a broad range of mechanisms of effect, liquorice is a plant that, in the future, may give rise to new therapies for pain.

## Introduction

**Figure 1.**
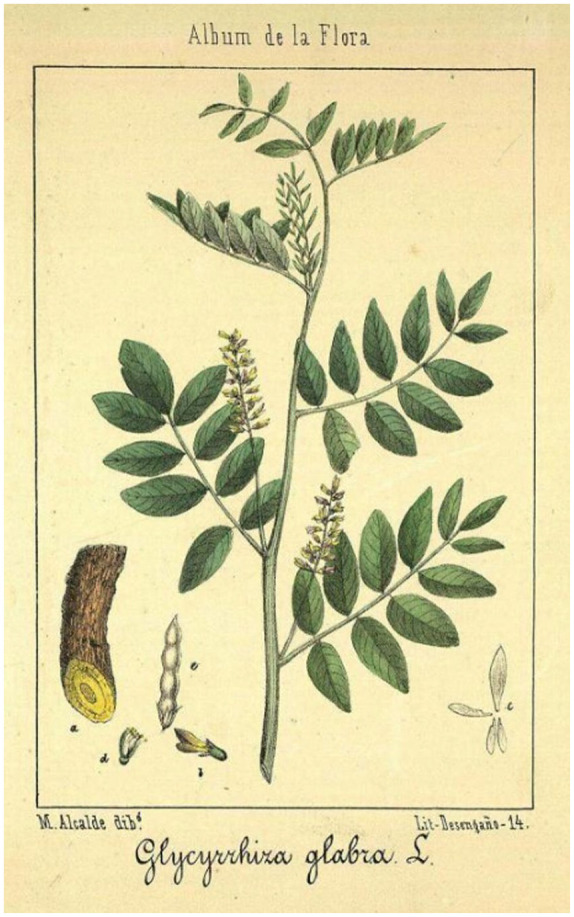
*Glycyrrhiza glabra*. L. M. Alcade illustrator Europeana source: Real Jardin Botánico Madrid Licensed with CC BY-NC-SA 4.0. To view a copy of this license, visit http://creativecommons.org/licenses/by-nc-sa/4.0/.

Liquorice has a long history of use in traditional Chinese (TCM), Ayurvedic and herbal medicine as a treatment for kidney, lung and liver ailments, gastric discomfort, arthritis and infections.^1,2^ Liquorice is also used in TCM to alleviate pain.^3^ In addition, the 10th edition of the European Pharmacopoeia contains monographs on liquorice root (*Liquiritiae radix*) and liquorice extract for flavouring purposes (*Liquiritiae extractum siccum ad saporandum*), a herbal drug and herbal preparation, respectively.^4^ The European Pharmacopoeia, the Committee on Herbal Medicinal Products of the European Medicines Agency and TCM use three plants as liquorice: *Glycyrrhiza glabra* L. (Figure 1), *Glycyrrhiza uralensis* Fisch. and *Glycyrrhiza inflata* Bat., with roots and rhizomes of the first two being used most commonly. All three plants contain numerous bioactive secondary metabolites. *Glycyrrhiza glabra* has the highest concentration of triterpenoids and *Glycyrrhiza uralensis* has the highest concentration of flavonoids.^5^ *Glycyrrhiza* species have been found to contain more than 20 triterpenes and 300 flavonoids, with 73 bioactive plant secondary metabolites being identified.^3^ A recent untargeted metabolomic analysis of the three liquorice species identified 101 significant metabolites.^6^

**Figure 2.**
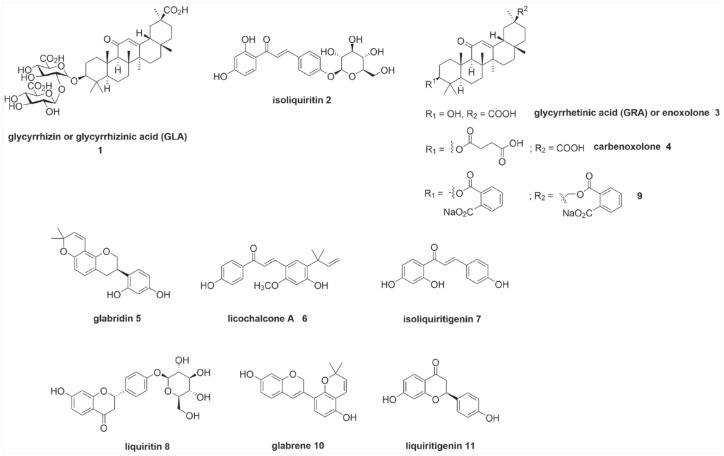
Terpenoids, flavonoids and chalcones from liquorice.

Glycyrrhizin (glycyrrhizinic acid, GLA, **1**) (Figure 2) is the main active compound of liquorice. GLA constitutes approximately 10% of the liquorice root dry weight and is present as a mixture of potassium, calcium and magnesium salts of GLA.^7^ GLA is a β-amyrin-type triterpenoid saponin, which numerous preclinical and cell studies report has antiviral, neuroprotective and potent anti-inflammatory properties.^3,8–11^ In Japan, GLA has been used as a medical drug in humans for more than 60 years to treat chronic hepatitis.^12^ Due to its antiviral and anti-inflammatory effects, GLA **1** was suggested as a treatment for severe acute respiratory syndrome (SARS),^13^ while most recently it has been designated a potential treatment for coronavirus disease 2019, COVID-19.^14,15^ Liquorice root constituents, including GLA **1** have also been shown to have anticancer effects and, since GLA **1** is both inexpensive and easily available, it has been proposed as a scaffold for the synthesis of new anti-tumour drugs.^16–18^

Liquorice root extract, its flavones and flavonones exert potent antioxidant effects, with lipid peroxidation inhibition being the most pronounced effect.^19^ For this reason, it is a source of antioxidants for the pharmaceutical, cosmetic and wellness industries.^19^

In TCM, liquorice is used to treat painful ailments. *Glycyrrhiza uralensis*, in combination in equal amounts with white peony root (*Radix paeoniae alba*), is a well-known Chinese herbal formula (Shaoyao-Gancao Decoction, Shakuyaku-Kanzo-to in Japanese), which is commonly used to relieve myalgia, arthralgia and neuropathic pain, including paclitaxel-induced pain.^20,21^ The liquorice root *O*-glycosidic *trans*-chalcone isoliquiritin **2** (Figure 2) has also been shown to have analgesic properties.^22^

This paper will explore the mechanisms by which glycyrrhizin **1**, the main active compound of liquorice, its major metabolite glycyrrhetic (glycyrrhetinic) acid (enoxolone, GRA **3**) and its synthetic analogue, carbenoxolone **4**, could influence nociception, analgesia, depression and morphine tolerance/ dependency. In addition, relevant actions of some of the major isoflavonoids isolated from liquorice root such as glabridin **5**, as well as the main *trans*-chalcones isoliquiritin **2** and licochalcone A **6**, the main *Glycyrrhiza trans-*chalcone (Figure 2) will be addressed. The compound structures with respective numbering are depicted in Figure 2 and a summary of their therapeutic actions and potential clinical use is portrayed in Table 1.

**Table 1.** Liquorice compounds, mechanisms of action and potential clinical use.

Compound (Numbers refer to the compound numbers in Figure 2)	Pharmacological group	Therapeutic actions	Potential clinical use
Liquorice root extract	Flavonoids, isoflavonoids, triterpenoids, chalcones as aglycones or their *O*-glucosides	• Antioxidant, lipid peroxidation ^19^ • Inhibition of opioid tolerance (α_2A_-adrenoceptor antagonism) ^56^	Postoperative sore throat* Laxative* Expectorant* Psoriasis & eczema*
Glycyrrhizinic acid, glycyrrhizin, GLA, **1**	Triterpenoid saponin	• Antiviral ^9,11^ • Neuroprotective ^8^ *(HMGB1 inhibition)* • Anti-inflammatory & antinociceptive ^23-37^ *(Cox-2 inhibition via NF-κB, PI3K, mPGE, 11β-HSD, HMGB1 inhibition)* • Inhibition of opioid tolerance and OIH ^54^ (*HMGB1 inhibition)* • Reduction of NP, anxiety and depression ^60-61^ (*inhibition of HMGB1*) • Anticancer ^16^	Chronic hepatitis C* SARS COVID-19 Scaffold for the synthesis of anticancer drugs Rheumatoid arthritis Endometriosis Ischaemic brain injury
Isoliquiritin, **2**	*trans*-Chalcone *O*-glucoside	• Antidepressant ^62-64^ (*increased 5-HT & NA*) • Antihyperalgesic, antiallodynic effects in NP ^22^ (*spinal 5-HT*)	Analgesia
Glycyrrhetic acid, glycyrrhetinic acid, enoxolone, GRA, **3** Carbenoxolone, 4**	Triterpenoid Semisynthetic triterpenoid	• Anti-inflammatory and anticancer ^24^ (*inhibition of COX-2 via 11β-HSD)* • Antinociception ^42^ (*tachykinin receptor antagonism)* • Lowering of intracellular cortisol and enhancement of insulin sensitivity ^71^ (inhibition of *11β-HSD)*	Diabetes type 2*
Glabridin, **5**	Isoflavane-type isoflavonoid	• Anti-inflammatory ^46^ • Antinociception ^46^ (*activation of BKCa channels, downregulation of NO and TRPV channels*)	
Licochalcone A, **6**	*trans*-Chalcone	• Anti-inflammatory ^31^ (*COX-2 inhibition*)	
Isoliquiritigenin, **7**	*trans*-Chalcone	• Anti-inflammatory ^30^ (*COX-2 inhibition*) • Antinociception ^45^ (*inhibition of Na_v_-channels*)	
Liquiritin, **8**	Flavanone *O*-glucoside	• Anti-inflammatory ^48^ (*downregulation of proinflammatory cytokines, upregulation of the anti-inflammatory cytokine IL-10*) • Antidepressant ^62,63^ (*increased 5-HT & NA*) • Antihyperalgesic, antiallodynic ^48^ (*inhibition of astrocyte and microglia activation, axon and myelin sheath repair*)	

11β-HSD = 11β-hydroxysteroid dehydrogenase; HMGB1, high mobility group box 1; NA, noradrenaline; NP, neuropathic pain, OIH, opioid-induced hyperalgesia; mPGE, microsomal prostaglandin E.

*Already in clinical use/positive RCTs

** licenced drug for Diabetes type 2 in the UK

## Liquorice: anti-inflammatory effects and mechanisms

A search of PubMed 10.01.21 using the terms ‘liquorice’ and ‘inflammation’ gave 318 hits, with a large body of preclinical literature reporting that GLA **1**, GRA **3** and other constituents of liquorice have potent anti-inflammatory properties and that they attenuate inflammation through a diverse range of mechanisms. In particular, the inhibitory effects of liquorice on cyclooxygenase-2 (COX-2), high mobility group box 1 protein (HMGB1) and gap junction/connexin function suggest that liquorice constituents may be of potential interest with regard to pain management.

Although a large number of triterpenes have been isolated from liquorice root, only GLA **1** and GRA **3** are reported to possess anti-inflammatory properties.^3^ Other liquorice root constituents such as glabridin **5**, licochalcone A **6**, isoliquiritigenin **7** and liquiritin **8** (Figure 2) have also been shown to exert anti-inflammatory effects.

### COX-2 inhibition

Many constituents of liquorice exhibit the ability to suppress COX-2.^23,24^ The triterpenoids GLA **1** and GRA **3** have anti-inflammatory effects, in part by suppressing the expression and activity of COX-2 through the inhibition of nuclear factor (NF)-κB and phosphoinositide-3-kinase (PI3K) activity.^25^ In a rodent study, magnesium isoglycyrrhizinate (MgIG) reversed methotrexate-induced hepatotoxicity.^26^ COX-2 expression was reduced significantly by 7 days administration of MgIG. In another preclinical study, a single dose of ammonium glycyrrhizinate induced anti-inflammatory and antinociceptive effects lasting 24–48 h, presumably due to its ability to bind the COX/microsomal prostaglandin E synthase (mPGE) pathway.^27^ A murine study found that GLA **1** had significant anti-inflammatory and antinociceptive activities that were mediated *via* attenuating the expression of COX-2 and proinflammatory cytokines.^28^ GLA **1** has been proposed recently as a treatment for rheumatoid arthritis and endometriosis by targeting COX-2.^23,29^

Both GLA **1** and GRA **3** are potent inhibitors of human 11-β-hydroxysteroid dehydrogenase (11β-HSD). Pharmacological inhibition of 11β-HSD type 2 (11β-HSD2) has been shown to decrease COX-2 expression and activity in colonic adenomas and tumours and significantly suppress adenoma and tumour growth, by increasing tumour glucocorticoid activity, which in turn selectively blocks local COX-2 activity.^24^ Glycyrrhizin **1** is an 11β-HSD2 inhibitor that has been shown to suppress adenoma COX-2 expression.^24^

The liquorice-derived phenolic *trans*-chalcones licochalcone A **6** and isoliquiritigenin **7** have both been found to suppress COX-2 expression.^30,31^

### HMGB1 inhibition

GLA **1** is a HMGB1 inhibitor. HMGB1 is a proinflammatory cytokine and the ligand for the receptor for advanced glycation end products (RAGE) and toll-like receptors, (TLR)-2 and TLR4.^32^ When HMGB1 is released to the extracellular space, it induces inflammation by enhancing pro-inflammatory signalling including the COX-2 pathway.^33^ HMGB1 also indirectly activates C-X-C chemokine receptor type 4 (CXCR4), which is involved in pain modulation in both the peripheral and central nervous systems, with increased receptor activation being associated with neuropathic pain and opioid-induced hyperalgesia.^34^ Animal studies suggest that HMGB1 plays a crucial role in neuroinflammation and the development of neuropathic pain. A recent study in diabetic rats and mice found that the inhibition of HMGB1-mediated inflammation by GLA **1** ameliorated diabetic neuropathy.^35^

In another rodent study, peripheral nerve injury [tibial nerve injury (TNI)] resulted in the redistribution of HMGB1 from the nucleus to the cytoplasm in sensory neurons.^36^ Systemic injection of GLA **1** reversed TNI-induced mechanical hyperalgesia at 14 days and 3 months following nerve injury, presumably through HMGB1 inhibition. Further support for the role of the HMGB1-TLR4 interaction was provided by Sun and colleagues who found that GLA **1** ameliorated inflammatory pain by inhibiting the microglial activation-mediated inflammatory response *via* blockage of the HMGB1-TLR4-NFkB pathway in murine BV2 microglial cells and a murine inflammatory pain model.^37^

HMGB1 inhibition seems to be one mechanism by which GLA **1** exerts neuroprotective effects, for example, in a rodent model of ischemic brain injury.^8^

### Gap junction/connexin blockade

Connexins are transmembrane proteins, many of which are expressed in the central nervous system, in neurons and glia. Connexins are key proteins for intercellular communication, forming hemichannels (connexons) and channels (gap junctions) that permit metabolic and functional communication between cells. Hemichannels formed by connexins are activated by proinflammatory cytokines and are important for the generation and maintenance of neuroinflammation.^38^ Spinal glial gap junctions appear to play an important role in pathologic pain conditions. Although GLA **1** does not affect gap junctions, carbenoxolone **4** – the synthetic analogue of its principal metabolite GRA **3** – is a gap junction blocker. Intrathecal **4** in rats following L5 spinal nerve ligation dose-dependently inhibited mechanical hypersensitivity at 2–3 weeks after the injury and attenuated heat hypersensitivity.^39^ A study which explored the analgesic potential of carbenoxolone **4**-induced connexin inhibition in a mouse model of cancer-induced bone pain found that chronic systemic administration of carbenoxolone **4** caused later onset and attenuation of movement-evoked and ongoing pain, assessed by limb use and weight bearing.^40^ The authors proposed that carbenoxolone **4** may prove a novel analgesic treatment for cancer-induced bone pain. In another preclinical study on bone cancer pain (BCP), intrathecal liquiritin **8** dose-dependently ameliorated bone cancer-induced mechanical allodynia and inhibited BCP-induced elevated astrocytic activation.^41^ In addition, liquiritin **8** inhibited BCP-induced chemokine (CXCL-1) upregulation in spinal astrocytes and attenuated BCP-induced chemokine (CXCR-2) upregulation in spinal cord neurons.^41^

### Other anti-inflammatory mechanisms

Further anti-inflammatory mechanisms of liquorice components have been described. Akasaka and colleagues examined the effects of GRA **3-**derived semisynthetic compounds on human tachykinin receptors expressed in CHO-K1 cells.^42^ In particular, the disodium salt derivative **9** (Figure 2) was found to suppress pain-related behaviour in the formalin test and reduced thermal hyperalgesia in the sciatic nerve injury model, through tachykinin receptor antagonism. Shaoyao-Gancao decoction which consists of peony root and liquorice root, has been shown in a rat model of arthritic pain to exert analgesic effect *via* downregulation of the TRPV1 channel.^43^ Liu *et al.* investigated the effects of systemic administration of GLA **1** in an Alzheimer mouse model and found that GLA **1** alleviated neuroinflammation by suppressing overexpression of proinflammatory cytokines in the mouse brain *via* inhibiting activation of the toll-like receptor 4 (TLR4) pathway.^44^ The liquorice-derived *trans*-chalcone isoliquiritigenin **7** was recently found to exert antinociceptive effects mainly through inhibitory action on voltage-gated sodium (Na_v_) channels on sensory nociceptive fibres.^45^ In a rodent model, the Iiquorice-derived isoflavane-type isoflavonoid glabridin **5** exhibited anti-inflammatory actions, inhibited cytokine production and showed anti-nociceptive response *via* activation of calcium-activated potassium (BKCa) channels and downregulation of NO levels and TRPV.^46^ Large conductance BKCa channels have been found to exert inhibitory control on sensory input in inflammatory pain.^47^ In a chronic constriction sciatic injury model in mice, intragastric liquiritin **8** dose-dependently reduced hyperalgesia and allodynia, restored the injured axon and myelin sheath, inhibited activation of astrocytes and microglia, downregulated the production of proinflammatory cytokines and simultaneously upregulated the anti-inflammatory cytokine interleukin 10.^48^

### Evidence for anti-inflammatory efficacy from clinical trials

Glycyrrhizin **1** has a long history of use for the treatment of chronic hepatitis B and C. A search of PubMed 10.01.21, using the terms ‘glycyrrhizin AND inflammation’ and the filter ‘randomised controlled trial’ gave only three relevant hits. All three trials included participants with chronic hepatitis C. Manns *et al.* conducted a phase III trial with 379 participants,^49^ and found that GLA **1** exhibited a significantly higher alanine transaminase (ALT) reduction compared with placebo after 12 weeks of therapy and improved necro-inflammation and fibrosis after 52 weeks of treatment. GLA **1** was generally well tolerated. Orient *et al.* included 72 participants in a randomised clinical trial and found that positive ALT responses induced by 4 weeks of GLA **1** therapy could be maintained in a subset of chronic hepatitis C patients receiving at least three injections weekly.^50^ However, the observed ALT response was not accompanied by significant histological improvement after 6 months of treatment. The third trial investigated combined treatment with GLA **1** and ursodeoxycholic acid in 170 participants, and concluded that the therapy was safe and effective in improving liver-specific enzyme abnormalities and could be an alternative to interferon in chronic hepatitis C virus infection, especially for interferon-resistant or unstable patients.^51^

GLA **1** in combination with glycine and L-cysteine, is available as the licenced drug Stronger Neo-Minophagen® C (SNMC). SNMC was used in Japan from 1948 to treat allergic disorders and was used for the treatment of chronic liver disease from 1958.^12^ It was approved in Japan for the treatment of liver function abnormalities in chronic liver disease in 1979.^12^ An additional search using the terms ‘SNMC OR Stronger Neo-Minophagen® C’ and the filter ‘randomised controlled trial’ yielded a further three trials, two of which involved SNMC in combination with other substances (*S*-adenosyl-L-methionine in one trial and decoction of turtle shell in the other). The remaining trial compared two doses of SNMC, administered three times a week, in patients with chronic viral hepatitis B or C, concluding that intermittent SNMC was efficient in lowering ALT levels, with only very mild adverse effects.^12^

Two clinical trials have reported that liquorice gargle reduces postoperative sore throat.^52,53^ The larger of these two trials had a randomised, double-blinded placebo-controlled design and included 236 participants intubated with a double lumen endotracheal tube in preparation for thorax surgery.^53^ The participants were randomised to one of two groups. At 5 min prior to induction of general anaesthesia, the intervention group gargled with liquorice solution (*Extractum liquiritiae fluidum;* liquorice 0.5 g) for 1 min. The control group followed the same procedure with simple syrup (*Sirupus simplex;* sugar 5 g). Using an 11-point Likert scale, pain at rest and on swallowing was assessed at several time points after arrival in the post anaesthesia care unit. Liquorice gargling halved the incidence of postoperative sore throat, a common complaint after endotracheal intubation.

## Reduction/inhibition of morphine tolerance and physical dependence

GLA **1** and derivatives appear to reduce/inhibit the development of morphine tolerance and physical dependence *via* several mechanisms. One possible mechanism is through HMGB1 inhibition. A recent study in rats found that morphine mediates upregulation of spinal HMGB1, contributing to analgesic tolerance and hyperalgesia *via* activation of TLR4/NF-κB signaling.^54^ The study also found that the HMGB1 inhibitor GLA **1** reduced morphine tolerance and alleviated morphine-induced withdrawal hyperalgesia. The authors concluded that GLA **1** may be a promising adjuvant to morphine in the treatment of intractable pain.

Another possible mechanism by which liquorice constituents could influence morphine tolerance is *via* α_2A_-adrenoreceptor antagonism. Low doses of competitive α_2_-adrenoreceptor agonists can potentiate acute morphine analgesia and block or reverse tolerance to spinal administration of morphine in experimental animals.^55^ Nakagawa *et al.* investigated the effects of a traditional Japanese medicine, Yokukansan (YKS), on morphine tolerance and physical dependence in mice.^56^ YKS consists of seven medicinal herbs, including the root and stolon of Chinese liquorice (*Glycyrrhiza uralensis* Fisch). The authors found that the membrane expression of α_2A_-adrenoreceptors in the pons/medulla of mice was decreased in morphine-withdrawn animals. Three compounds of Chinese liquorice in YKS, namely isoliquiritin **2**, isoliquiritigenin **7** and glycyrrhetic acid **3**, had specific binding affinity for and antagonist activity against the α_2A_-adrenoreceptor. Repeated administration of Chinese liquorice extract and its active substance GLA **1** inhibited morphine withdrawal signs significantly.^56^

## Antidepressant effects

The immune system appears to play a role in the pathology of psychiatric disorders and there is clear evidence of correlations between immune dysfunction and psychiatric disease.^57^ The immune system engages directly with central nervous system glia,^58^ and neuroinflammation can be caused by excessive production of proinflammatory cytokines in microglia, which are the resident immune cells of the brain. HMGB1 acting directly on microglia appears to be a major mediator in various neuroinflammatory diseases. HMGB1 has been shown to mediate depressive behaviour induced by chronic stress through activating the kynurenine pathway.^59^ GLA **1** blockage of HMGB1 in partial sciatic nerve ligation (PSNL) mice reduced microglia activation and anxiodepressive-like behaviour.^60^

A recent clinical study found that GLA **1** as an adjuvant to selective serotonin reuptake inhibitors (SSRI) had better effect in depressed patients than SSRI and placebo and that the effect was more pronounced in those who had a higher grade of inflammation [cut-off concentration C-reactive protein (CRP) ⩾3 mg/l].^61^ While acknowledging the limitations of the study due to small size and short duration, the authors proposed that patients with depression should be classified according to grade of inflammation and that those with a high grade of inflammation should receive adjuvant treatment with an anti-inflammatory agent, such as GLA **1**.

The liquorice plant-derived *O*-glycosidic *trans*-chalcone isoliquiritin **2** and *O*-glycosidic flavanone liquiritin **8** have been found to have antidepressant-like effects in rodent models,^62,63^ with the effects most likely due to increased serotonin and noradrenaline in the hippocampus, hypothalamus and cortex.^62,64^ In addition, isoliquiritin **2** appears to have antihyperalgesic and antiallodynic effects in neuropathic pain. A murine study demonstrated these effects and found them to be dependent on spinal serotonergic mechanisms.^22^

## Current clinical use of liquorice

The liquorice plant has a long tradition of use in traditional Chinese medicine and has also been used extensively in herbal medicine and folk remedies in other countries.^7^ Liquorice is commonly used in modern medicine as a laxative and as an expectorant in cough syrups. There are a number of clinical studies documenting long-term therapy with GLA **1** for chronic hepatitis, due to its anti-viral, anti-inflammatory and hepatoprotective effects,^65^ with it recently being proposed as a possible secondary chemoprevention strategy for hepatocellular carcinoma.^66^ GLA **1** is also used as oral, intravenous or topical adjuvant therapy for dermatological disorders such as psoriasis and eczema.^67,68^

Carbenoxolone **4** is a licenced drug in the United Kingdom (UK). GRA **3** is reported to be 200–1000 times more potent inhibitor of 11β-HSD, compared with GLA **1.**^69^ Carbenoxolone **4** was used primarily to accelerate the healing of gastric and duodenal ulcers,^70^ and is currently a therapeutic agent for diabetes 2, due to its inhibitory effect on 11β-HSD type 1, with the effect of lowering intracellular cortisol concentrations and enhancing insulin sensitivity.^71^ It should be noted that carbenoxolone **4** also blocks 11β-HSD type 2 and binds to mineralocorticoid receptors as an agonist inducing pseudo-hyperaldosteronism.^71^

## Liquorice: other uses

Apart from pharmaceuticals, GLA **1** is used as a sweetener and flavouring agent in the confectionery,^2^ food and tobacco industry. It was approved for use as a food additive in 1985 in the United States (US) and has been given the status of Generally Recognized as Safe (GRAS) by the US Food and Drug Administration (FDA).^14^ Glycyrrhizin **1** is reputed to be approximately 50 times sweeter than sucrose and has no effect on the glycaemic index; however, its use as a sweetener is limited by the intense liquorice flavour. The isoflavane-type isoflavonoid glabridin **5** is used by the cosmetic industry in antioxidant and anti-inflammatory creams and as a whitening agent. Liquorice plant extracts are also used as a feed additive for livestock and poultry, due primarily to their immunomodulatory, anti-inflammatory and antiviral effects.^72^

## Adverse effects

The scientific literature contains many case reports on the adverse effects of liquorice, most recently a report of cardiac arrest due to liquorice-associated mineralocorticoid excess with profound hypokalaemia,^73^ hypertension and hypokalaemia attributed to chronic ingestion of herbal tea containing liquorice root and hypokalaemia attributed to liquorice-flavoured vaping.^74,75^ The adverse effects of liquorice seem to be related to dose, duration and individual susceptibility and are usually reversed after discontinuation of liquorice. It has long been established that liquorice can cause potentially serious dose-dependent adverse effects in the form of pseudohyperaldosteronism (high blood pressure, sodium retention, hypokalaemia and oedema). This is due to GLA **1**/GRA **3**-related inhibition of 11β-HSD2 – the enzyme that converts the active cortisol to locally inactive cortisone – leading to increased levels of cortisol with activation of renal mineralocorticosteroid receptors. A systematic review found that chronic consumption of liquorice, or products containing GLA **1**, in healthy subjects increases systolic and diastolic blood pressure and decreases plasma potassium levels, concluding that these changes, while modest, may be particularly relevant for persons with pre-existing cardiovascular or renal disease.^76^ GLA **1** is known to be a potent inhibitor of placental 11β-HSD2 and may lead to higher levels of foetal cortisol exposure. An epidemiological study found that high maternal consumption of GLA **1** in pregnant women may adversely affect the developing offspring.^77^

Considering the potential for adverse effects, the long half-life of glycyrrhizin **1** and the enterohepatic recirculation (see below), the efficacy and safety of intermittent dose regimens should be considered. According to one author, when treating gastrointestinal inflammation with carbenoxolone **4**, the common dosage is ‘100 mg for 1 week, three times a week, followed by a reduction to 50 mg every other day, to prevent potential side effects such as sodium retention, hypokalaemia, increased blood pressure and edema’.^78^

Both GLA **1** and GRA **3** exhibit antithrombotic properties, inhibiting coagulation factor Xa and thrombin. A case of haemorrhagic stroke and cerebral microbleeds attributed to high intake of liquorice root supplement has been reported.^79^

Liquorice also has endocrinological properties, with oestrogen-like effects due mainly to the root isoflavonoids glabrene **10** (Figure 2) and glabridin **5** as well as *trans*-chalcone isoliquiritigenin **7**, while the triterpenoids GLA **1** and GRA **3** exhibit weak anti-androgen effects.^7^ A study in nine healthy women found that daily ingestion of 3.5 g of commercially prepared liquorice for 2 months resulted in slight, but statistically significant, increases in serum parathyroid hormone, 25-hydroxycholecalciferol and urinary calcium levels, compared with baseline.^80^ All parameters returned to baseline values by 1 month after discontinuation of liquorice. The same group found that total testosterone is reduced slightly in healthy males and females after 1 week of daily liquorice intake.^81,82^

The maximum dose of GLA **1** recommended by the European Commission Scientific Committee on Food is 100 mg per day, although the committee acknowledged the limited amount of research on human toxicity of this compound.^83^

A comprehensive review on the toxicology of liquorice, which acknowledged the difficulty of assessing safety due to the differences in the administered substances tested, estimated that, based on rodent studies, a no-observed-effects-level (NOEL) for purified GLA **1** would be in the range of 15–229 mg/kg/day and, by applying uncertainty factors for intraspecies and interspecies differences, an acceptable daily intake (ADI) for GLA **1** would be 0.015–0.229 mg/kg/day.^84^ This estimate is in line with a previous toxicological assessment of an ADI for GLA **1** of 0.2 mg/kg/day.^85^

## Pharmacokinetics

Studies in rodents and humans have demonstrated that GLA **1** is poorly absorbed by the gastrointestinal tract, but extensively metabolized by the intestinal microflora to GRA **3** and monoglucuronyl glycyrrhetic (glycyrrhetinic) acid, both of which are readily absorbed. GLA **1** and GRA **3** pharmacokinetics are characterised by a biphasic elimination from the central compartment with a dose-dependent second elimination phase.^86^ An enterohepatic recycling of glycyrrhetinic acid **3** can occur, requiring several days for complete elimination.^87^

A pharmacokinetic study in three healthy subjects found that, after oral administration of 100 mg of GLA **1**, the major metabolite GRA **3** appeared in plasma.^88^ However, GLA **1** corresponding to 1.1–2.5% of the oral dose was found in urine, suggesting that GLA **1** is at least partially absorbed intact from the gastrointestinal tract. When GLA **1** at 40, 80 or 120 mg was administered intravenously, its concentration in plasma showed biexponential profiles during the 24 h after administration, but GRA **3** and glycyrrhetic acid-3-*O*-glucuronide were not detected in either plasma or urine. Three dosing experiments found that the terminal half-life of GLA **1** was 2.7–4.8 h, the apparent volume of the central compartment was 37–64 ml/kg, the steady-state distribution volume was 59–98 ml/kg and the total body clearance was 16–25 ml/kg/h. GLA **1** was not detected in plasma after oral administration of the usual therapeutic dose and no dose dependency was observed in the dose range of 40–120 mg.^88^

The constituents of liquorice have complex effects on CYP enzymes, and further research is required to elucidate these effects.^89^ For example, liquorice extract has been shown to be a potent inhibitor of CYP3A4 activity. This effect appears to be due not to the major active component GLA **1** but to other constituents of the extract, such as flavanone-class liquiritigenin **11** (Figure 2).^89^ Both GLA **1** and GRA **3** appear to increase CYP3A4 activity, whereas GRA **3** and isoflavane-class glabridin **5** inhibit CYP2C9. Liquorice may therefore potentiate the effects of warfarin therapy.^89^

GLA **1** has been shown to have effects on the pharmacokinetics of prednisolone. A pharmacokinetic study in six healthy men treated with intravenous prednisolone (prednisolone hemisuccinate, PSL-HS) found that pretreatment with oral administration of GLA **1** significantly increased the concentrations of total prednisolone (PSL) at 6, 8 h, and of free PSL at 4, 6 and 8 h after PSL-HS infusion.^90^ Oral administration of GLA **1** was also found to modify the pharmacokinetics of both total and free PSL. The authors concluded that oral GLA **1** increases plasma prednisolone concentration and influences its pharmacokinetics by inhibiting its metabolism, but without affecting its distribution.

## Potential for new drug development

A recent paper by Thomford and colleagues addressed the challenges involved in evaluating and using plant products and the difficulty in accepting their therapeutic efficacy.^91^ The authors acknowledged the difficulty of assessing complex molecular mixtures such as liquorice, emphasising that the therapeutic activity of plant extracts is usually because of the synergistic and simultaneous action of several constituents, rather than single compounds. Design and synthesis of novel semisynthetic derivatives, analogues and mimetics of GLA **1** and other constituents of *Glycyrrhiza* species will be mandatory to produce libraries of chemically purified single compounds from such extracts for additional biological and pharmacological studies of plants belonging to the bean family (Fabaceae).

## Conclusions

Liquorice plant and its herbal preparations have had centuries of use in traditional Chinese, Ayurvedic and herbal medicine for a range of ailments, including pain. Liquorice constituents have been found to have anti-inflammatory, antioxidant, antiviral, anticancer, hepatoprotective and neuroprotective properties. In addition, they appear to have antidepressant actions and effects on morphine tolerance. Liquorice constituents such as glycyrrhizin (GLA) **1**, its metabolite glycyrrhetic (glycyrrhetinic) acid (GRA) **3** and other liquorice-derived compounds such as glabridin **5** and isoliquiritigenin **7** exert these effects *via* a range of mechanisms including HMGB1 inhibition, gap junction blockade and α_2A_-adrenoceptor antagonism. A large body of preclinical studies and a long history of use in herbal medicine suggest that liquorice constituents may be useful for pain management. However, whether liquorice plant-derived compounds represent a novel class of analgesics is yet to be established. Regular administration of liquorice can cause serious adverse effects and this could prove a limiting factor with regard to chronic pain management. Clinical research examining available preparations such as liquorice root extract or single compounds such as glycyrrhizin **1**, carbenoxolone **4**, liquiritin **8**, isoliquiritigenin **7**, isoliquiritin **2**, or glabridin **5** or new liquorice-derived drugs in clearly defined models of acute and chronic pain, with regard to efficacy, tolerability and safety are required. Having a host of bioactive compounds with a broad range of mechanisms of effect, liquorice is a plant, which in the future may give rise to new therapies for pain.
